# Expression and clinical significance of miR-4516 and miR-21-5p in serum of patients with colorectal cancer

**DOI:** 10.1186/s12885-020-06715-6

**Published:** 2020-03-23

**Authors:** Xi-Han Jin, Sen Lu, Ai-Fen Wang

**Affiliations:** 1 Department of Colorectal Surgery, Jinhua Hospital of Zhejiang University, Jinhua Municipal Central Hospital, Jinhua, 321000 China; 2 Department of Colorectal Surgery, The First Affiliated Hospital, College of Medicine, Zhejiang University, Hangzhou, 310003 China; 3 Department of Oncology, The First Hospital of Jiaxing, The First Affiliated Hospital of Jiaxing University, No. 1882 South Zhonghuan Road, Nanhu District, Jiaxing, 314001 China

**Keywords:** Colorectal cancer, miR-4516, miR-21-5p, Serum

## Abstract

**Background:**

This study sought to detect the expression and clinical significance of miR-4516 and miR-21-5p in serum of patients with colorectal cancer.

**Methods:**

Bioinformatics methods were used to analyze the expression patterns of miR-4516 and miR-21-5p in colorectal cancer. A total of 80 patients with colorectal cancer, 65 patients with benign colorectal tumors and 50 healthy persons were selected. qRT-PCR was performed to detect the expression levels of serum miR-4516 and miR-21-5p before and after operation or postoperative recurrence. The correlation of miR-4516 and miR-21-5p expression levels with the clinical characteristics and prognosis of colorectal cancer was analyzed, and that with the patient’s survival was further examined by Kaplan-Meier analysis.

**Results:**

MiR-4516 was poorly expressed in colorectal cancer in the preoperative group, and miR-21-5p was highly expressed. While in the postoperative group, miR-4516 was up-regulated, and miR-21-5p was down-regulated. The low expression of miR-4516 was shown to be related to TNM staging, invasion degree, lymph node metastasis and distant metastasis of the patients. Whereas the high expression of miR-21-5p was proved to be correlated with TNM staging and lymph node metastasis. Kaplan-Meier survival analysis showed that high expression of miR-4516 or low expression of miR-21-5p could contribute to better overall survival.

**Conclusion:**

Low miR-4516 or high miR-21-5p could be used as an independent risk factor for prognosis of colorectal cancer.

## Background

Colorectal cancer (CRC) is the third most common malignancy in the world, including colon cancer and rectal cancer, with the incidence and mortality in China annually increased [1]. Although many factors like environmental factors (such as high-fat diet, intestinal flora imbalance), genetic factors and inflammatory bowel disease can lead to CRC, about 80% of CRC cases are developed from colorectal adenoma (CRA). In addition, despite the great advance in current technology for CRC treatment, the response on metastatic CRC is still very poor, which makes the early diagnosis of CRC become the key for mortality reduction and prognosis improvement [2, 3]. Therefore, the search for a more sensitive, easy to be detected and representative biomarker is of great significance for CRC early diagnosis and monitoring.

MicroRNAs (miRNAs) are evolutionarily conserved, endogenous and non-coding small RNAs playing a key role in the regulation of gene expression in key cellular processes [4, 5]. Studies have shown that miRNAs participate in a series of life processes acting as important regulators during tumorigenesis and development, including individual development, organogenesis, hematopoiesis, cell proliferation, differentiation and apoptosis, tumor occurrence, invasion and metastasis [6, 7]. miRNAs are available in a non-invasive manner with high sensitivity (62 to 89%) and specificity (from 41 to 89%), and they can be used to identify CRC patients with normal persons [8]. miR-21 is one of the miRNAs found by human earlier, and it can be highly expressed in many cancer types, such as pancreatic cancer, breast cancer, lung cancer, stomach cancer and CRC [9–11]. Meanwhile, miR-21 is involved in the characteristics regulation of tumor phenotypes, like tumor growth, invasion and metastasis. miR-4516 can promote cell apoptosis [12], predict the poor prognosis of human glioblastoma [13], and targeted regulate psoriasis [14], but its role in CRC has not been studied. Recent studies have shown that miRNAs are abnormally expressed in peripheral blood of CRC patients, and they are involved in CRC progression with the potential serving as biomarkers [15].

In this study, we detected the expression of miR-21-5p and miR-4516 in tissues and serum of patients with CRC, CRA and healthy persons, analyzed the relationship between their expression levels and clinicopathological characteristics, and explored the value of miRNAs in serum in the early diagnosis of CRC.

## Methods

### Research object

80 patients who were pathologically diagnosed as CRC and treated in our hospital from January to October 2016 were selected as CRC group, including 38 males and 42 females aged 29 to 75 years old with an average of 50.90 (± 9.50) years old. Inclusion criteria: 1. In accordance with the clinical diagnostic criteria of CRC; 2. Patients newly diagnosed as CRC; 3. Had not receive any radiotherapy, chemotherapy or biological therapy. Exclusion criteria: 1. With other types of tumor diseases; 2. Postoperative patients or those with CRC recurrence; 3. With severe functional diseases like heart, liver, kidney diseases and immune system diseases; 4. Being treated with other therapies before surgery; 5. Patients in pregnant; 6. Patients who did not comply with, and cooperate with the treatment. Totally 65 patients with colon polyp in our hospital were selected as benign colorectal tumor group (CRA group), including 30 males and 25 females aged 30 to 71 years old with an average of 44.45 (± 10.94) years old. They were all pathologically diagnosed as low-grade intraepithelial neoplasia or colon hyperplastic polyp after operation. In addition, 50 cases who made physical examination in our hospital were selected as healthy control group (Normal group), including 28 males and 22 females aged 28 to 72 years with an average of 42.32 (±9.23) years old, and there were no abnormalities observed by colonoscopy. There were no significant differences in gender, age and other general data among the three groups (*P* > 0.05). Sample and relevant data collection in this study was approved by the Medical Ethics Committee, and the relevant informed consent was obtained from all patients.

### Main reagents

Trizol total RNA extraction kit was purchased from Beijing Aidlab Biotechnologies Co., Ltd.. Fully automatic electrochemiluminescence immunoassay (Model Number: E2010) was from Roche, Switzerland and high-speed refrigerated centrifuge was procured from Eppendof, Germany.

### Methods

#### Specimen collection

6 mL of venous blood were drawn from all enrolled patients with an empty stomach in the morning, respectively, and collected in vacuum blood collection tubes for 30 min at room temperature, then centrifuged at 2500 r/min for 10 min. The collected serum was stored in a refrigerator at − 80 °C.

#### Real-time quantitative PCR (qRT-PCR)

Trizol Total RNA Extraction Kit was used to extract the total RNA from serum. 5 μl of RNA samples were diluted 20 fold with RNase-free ultrapure water, and the absorbance values at 260 nm and 280 nm were read using a UV spectrophotometer for the determination of the RNA concentration and purity. RNA samples with 1.7 ≤ OD260/OD280 ≤ 2.1 were qualified for subsequent analysis. PCR amplification instrument was used to synthesize the cDNA template by reverse transcription reaction, and ABI7500 quantitative PCR instrument was applied to perform qRT-PCR. The reaction conditions were pre-denaturation at 95 °C for 10 min, and 35 PCR cycles (95 °C for 15 s, 60 °C for 1 min). U6 was utilized as internal reference. The primers used in the reaction were as follows:

- miR-4516
- Forward Primer: 5′-CCGCCGACTAGTGCGGATCATAAGGTCAGGAG-3′
- Reverse Primer: 5′-CCGCCGACGCGTGGCCAGGACAGCAGCTTA − 3′
- miR-21-5p
- Forward Primer: 5′-GCCCGCTAGCTTATCAGACTGATG-3′
- Reverse Primer: 5′-GTGCAGGGTCCGAGGT-3′

#### Detection of serum CEA

Automatic electrochemiluminescence immunoassay was used for detection the serum CEA. According to the CEA kit, the diagnostic critical reference value of CEA was 5 ng/mL.

#### Follow-up

Postoperative patients of the CRC group came to our hospital to review the CEA and perform abdominal CT scan after 0, 6, 12, 18, 21 and 24 months, respectively. Combined with the clinical symptoms of the patients, we could determine whether there was local recurrence or metastasis. Blood samples of patients with recurrence or metastasis were collected. The follow-up period was 2 years.

### Statistical analysis

All data were processed by SPSS 22.0 statistical software. The measurement data were expressed in the form of mean value ± standard deviation. The count data were presented as a ratio or percentage. The comparison of measurement data was performed by *t* test, and that of count data was performed by χ2 test. ROC curve was utilized to analyze the efficacy of indicators in the diagnosis of each group. Kaplan-Meier survival analysis was used to analyze the relationship between miR-4516 and miR-21-5p expression levels and postoperative survival in CRC patients. *P* < 0.05 was considered statistically significant. *P* < 0.01 was considered extremely statistically significant.

## Results

### The expression levels of miR-4516 and miR-21-5p in serum

**Fig. 1:**
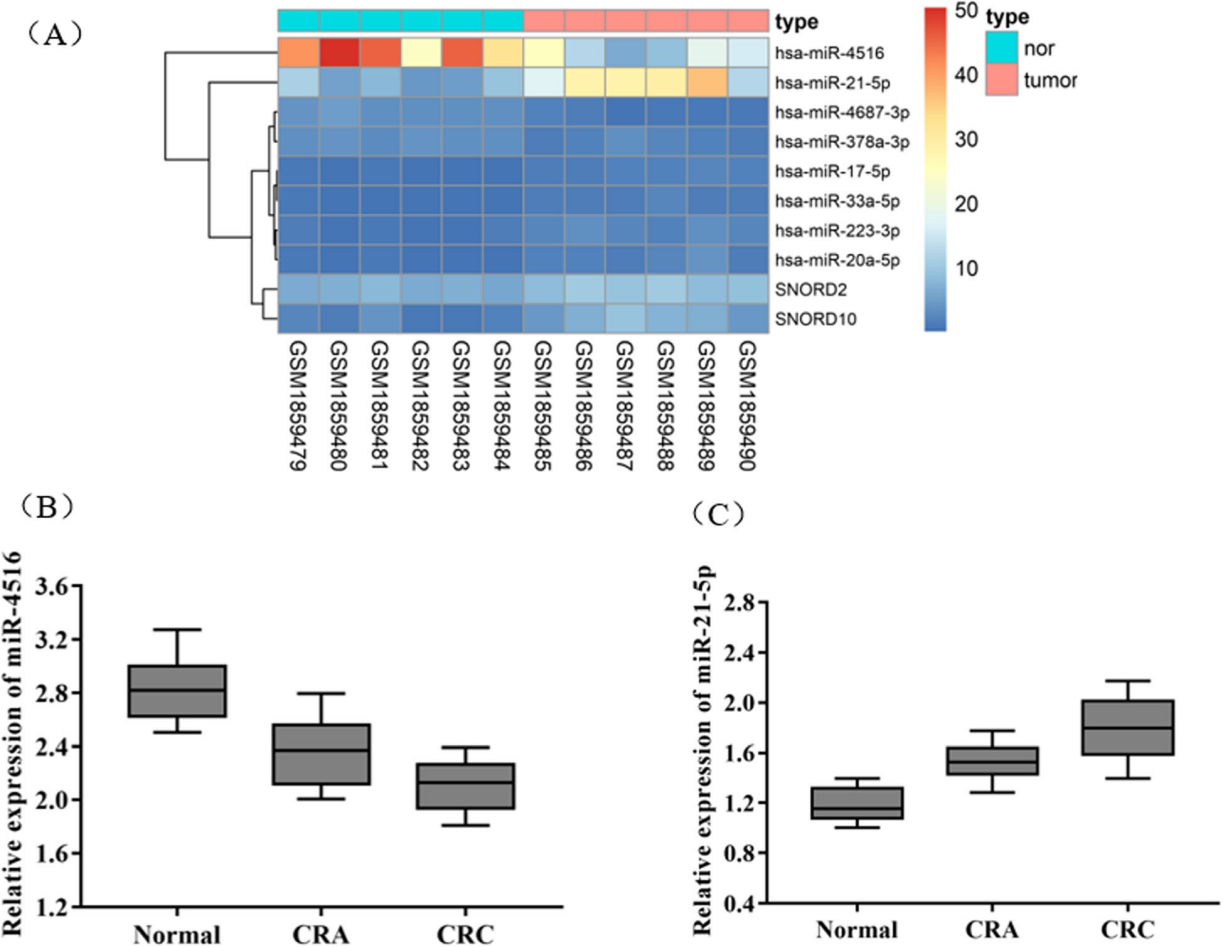
Expression levels of serum miR-4516 and miR-21-5p in CRC. **a** Cluster thermogram of the differentially expressed miRNAs in CRC detected via bioinformatics analysis; **b** miR-4516 expression level tested by qRT-PCR; (C) miR-21-5p expression level examined by qRT-PCR

The differential expression of miRNAs in colon cancer was analyzed using the GSE72281 chip in the GEO database. As shown in Fig. 1a, miR-4516 was poorly expressed in colorectal cancer, while miR-21-5p was highly expressed. Then, qRT-PCR was used to detect the expression levels of miR-4516 and miR-21-5p in the serum of the Normal group, CRA group and CRC group. The results shown in Fig. 1b and Fig. 1c suggested that miR-4516 was significantly down-regulated (*P* < 0.05) in the CRA group and CRC group relative to the Normal group, whereas miR-21-5p was significantly up-regulated (*P* < 0.05).

### Postoperative dynamic changes of serum miR-4516 and miR-21-5p in the CRC Group

**Fig. 2:**
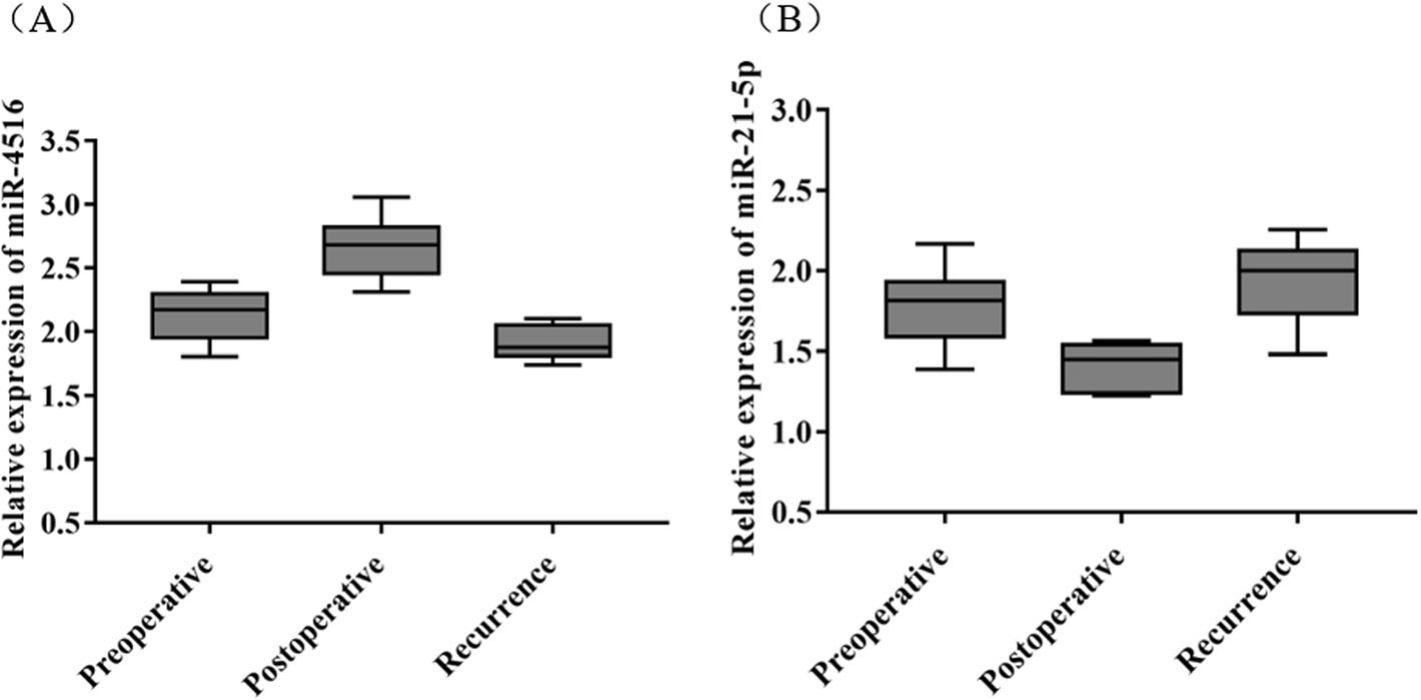
Dynamic changes of serum miR-4516 and miR-21-5p in CRC. **a** miR-4516 expression level detected by qRT-PCR; **b** miR-21-5p expression level assayed by qRT-PCR

After following-up, recurrence occurred in 18 patients in the CRC group, including local recurrence in 12 cases and distant metastasis in 6 cases. As shown in Fig. 2, serum miR-4516 in the CRC group was significantly up-regulated after operation while miR-21-5p was remarkably down-regulated (*P* < 0.05). There was no significant difference observed in miR-4516 and miR-21-5p expression levels between the preoperative and recurrence groups (*P* > 0.05).

### Relationship between the serum miR-4516 and miR-21-5p expression levels and Clinicopathological parameters of CRC

As shown in Table 1, the low expression of miR-4516 was related to TNM staging, invasion degree, lymph node metastasis and distant metastasis, with statistical significance (all *P* < 0.05). While the high expression of miR-21-5p was shown to be correlated with TNM staging and lymph node metastasis, with statistical significance (both *P* < 0.05).

**Table 1:** Relationship between the expression of miRNAs and clinicopathological characteristics of colorectal cancer

Group	*N* = 80	miR-4516		miR-21-5p	
		Relative Expression Level [M (*P*_25_, *P*_75_)]	*P*	Relative Expression Level [M (*P*_25_, *P*_75_)]	*P*
Age					
< 60	43	2.09 (1.89, 2.25)	0.0969	1.75 (1.54, 2.01)	0.2768
≥ 60	37	2.08 (1.95, 2.16)		1.83 (1.65, 2.03)	
Gender					
male	38	2.14 (1.95, 2.24)	0.8576	1.74 (1.55, 1.96)	0.1522
female	42	2.11 (1.96, 2.27)		1.84 (1.64, 2.04)	
Tumor Size					
< 5 cm	35	2.10 (1.96, 2.26)	0.7774	1.74 (1.53, 1.94)	0.4806
≥ 5 cm	45	2.04 (1.95, 2.24)		1.80 (1.62, 1.96)	
Tumor Location					
colon	38	2.12 (1.97, 2.29)	0.5136	1.68 (1.53, 1.90)	0.4000
rectum	42	2.15 (1.99, 2.22)		1.78 (1.55, 1.96)	
Infiltration Degree					
T1,T2	30	1.94 (1.88, 1.98)	0.0432	1.76 (1.59, 1.92)	0.0758
T3,T4	50	2.17 (2.07, 2.26)		1.79 (1.54, 1.94)	
TNM Stage					
I-II	44	2.40 (2.20,2.48)	< 0.001	2.04 (1.90,2.19)	0.001
III-IV	36	1.79 (1.74, 1.92)		1.58 (1.35, 1.64)	
Lymph Node Metastasis					
non	48	2.33 (2.14, 2.39)	0.002	2.04 (1.90,2.19)	0.003
exist	32	1.80 (1.82, 1.90)		1.58 (1.35, 1.64)	
Distant Metastasis					
non	52	2.37 (2.20, 2.42)	0.029	1.64 (1.50, 1.88)	0.301
exist	28	1.81 (1.74, 1.89)		1.77 (1.58, 1.96)	
CEA Level (ng/ml)					
< 5	41	2.09 (1.97, 2.20)	0.417	1.88 (1.68, 1.92)	0.064
≥ 5	39	2.12 (1.99, 2.24)		1.60 (1.59, 1.79)	

### ROC analysis of serum miR-4516 and miR-21-5p in CRC diagnosis

**Fig. 3:**
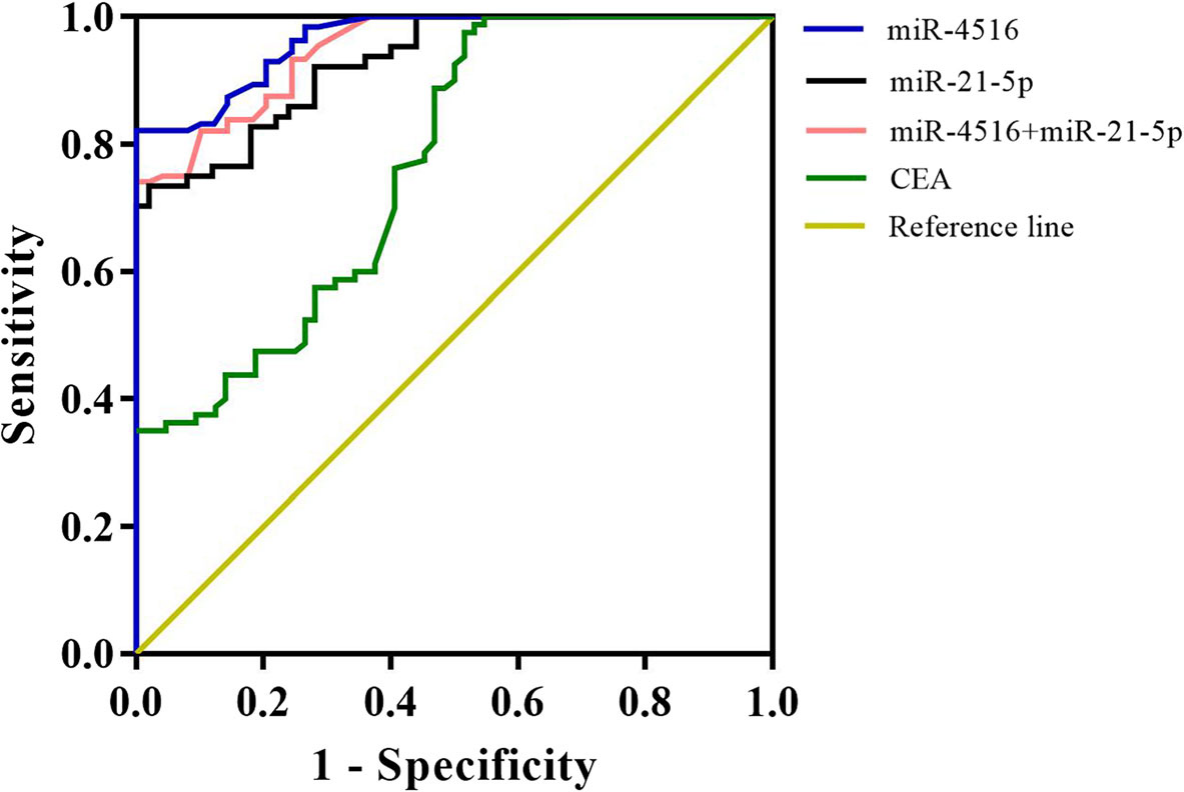
ROC analysis of serum miR-4516 and miR-21-5p in CRC

To further evaluate the value of miR-4516 and miR-21-5p in identifying CRC patients from normal persons, we performed ROC analysis in 80 CRC patients, and the results were shown in Table 2. The ordinate refers to the sensitivity and the abscissa refers to the specificity. ROC curves were made as Fig. 3, and the areas under the curve (AUC) were shown in Table 2. The AUC values were tested, and all of them have *P* < 0.05. When the Cut-off reached the optimal value, it could be concluded that the diagnostic efficiency of miR-4516, miR-21-5p, and the combination of miR-4516 and miR- 21-5p in CRC were better than that of CEA.

**Table 2:** Analysis on the expression levels of miR-4516 and miR-21-5p detecting the ROC of colorectal cancer

Variate	Sensitivity (%)	Specificity (%)	AUC	*P* Value	Cut-off
miR-4516	94.40	89.80	0.9584	< 0.001	2.17
miR-21-5p	90.63	86.20	0.9278	< 0.001	1.53
CEA	85.70	84.90	0.774	< 0.001	1.20
miR-4516 + miR-21-5p	92.11	87.60	0.9425	< 0.001	1.88

### Relationship between the serum miR-4516 and miR-21-5p and prognosis of CRC patients

**Fig. 4:**
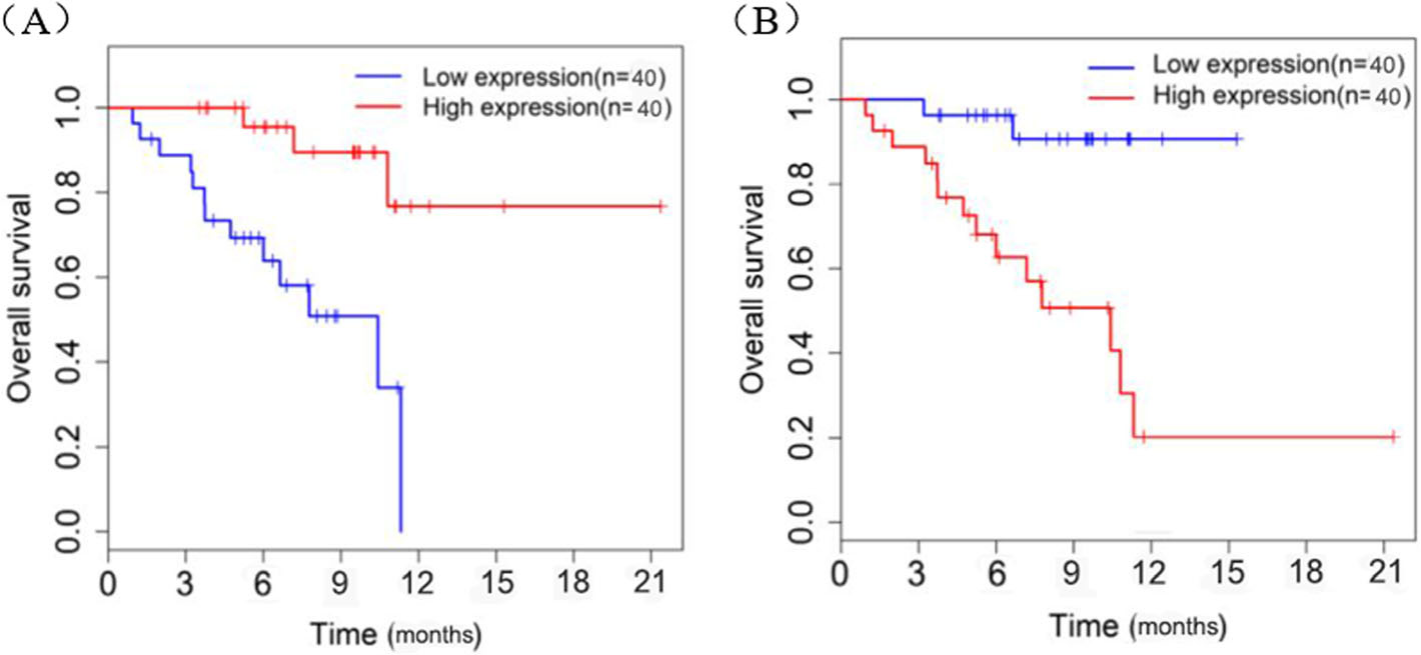
Relationship between the serum miR-4516 and miR-21-5pexpression levels and Prognosis of CRC Patients. **a** Kaplan-Meier curve of miR-4516; **b** Kaplan-Meier curve of miR-21-5p

Kaplan-Meier survival analysis was performed on the miR-4516 and miR-21-5p in CRC patients (Fig. 4). The results revealed that high miR-4516 or low miR-21-5p could significantly increase the overall survival rate, and the differences were statistically significant (both *P* < 0.05).

## Discussion

Colon cancer is one of the most common malignancies in the world. The occurrence of colon cancer is a very complex pathogenic process that is controlled by multiple genes and formed in different stages over a long period of time [16–18]. With the development of molecular biotechnology, miRNAs related to colon cancer have been discovered one after another. Studies have shown that more miRNAs are discovered to be present in colon cancer and play an important role in the tumorigenesis, development and prognosis. We used the colon cancer tissue chip GSE72281 in the GEO database to explore the potential role of miRNAs in colon cancer and found that miR-21-5p and miR-4516 are differentially expressed in the tissue chip. In recent years, it has been reported that miR-21 has a close relationship with colon cancer, and acts as a key molecule in EMT development. Besides, miR-21 overexpression is controlled by both genetic and epigenetic alterations, and its levels can be used as a tool for predicting CRC metastasis [9, 11]. However, miR-4516 is found in the serum of esophageal squamous cell carcinoma and differentially expressed [19]. Blood samples are easy to obtain, and the detection of serum miRNAs for CRC diagnosis and prognosis prediction is more suitable for clinical application. However, reports on miR-4516 in colon cancer serum are rare. Therefore, we used serum samples to explore the significance of miR-4516 and miR-21-5p expression and clinical diagnosis of colorectal cancer patients.

In this study, qRT-PCR was used to detect the expression levels of miR-4516 and miR-21-5p in serum of 80 CRC patients, 65 CRA patients and 50 normal persons. The results showed that miR-21-5p was highly expressed in serum of CRC patients, which is consistent with the literature [11]. Besides, serum miR-4516 and miR-21-5p in CRC patients changed dynamically after surgery and recurrence, indicating that both miR-4516 and miR-21-5p may have diagnostic value for CRC. In addition, correlation analysis was conducted and found that miR-4516 was significantly associated with TNM staging, invasion degree, lymph node metastasis and distant metastasis, whereas miR-21-5p showed great correlation with only TNM staging and lymph node metastasis, which suggested that both miR-4516 and miR-21-5p may act as oncogenes responsible for the CRC progression with diagnostic value. Moreover, ROC and Kaplan-Meier analyses indicated that miR-4516 and miR-21-5p could be used as an auxiliary diagnostic criterion for judging the CRC prognosis.

As serum is easy, fast to obtain and non-invasive, and miRNAs in serum are stable, serum miRNAs can be used as novel biomarkers to assist in the CRC diagnosis with good clinical significance, which makes early screening and diagnosis of CRC possible. The alterations of miR-4516 and miR-21-5p in serum suggest that these two miRNAs have the potential serving as tumor markers, and can be used as new therapeutic targets in CRC treatment.

## Data Availability

The data used to support the findings of this study are included within the article. The data and materials in the current study are available from the corresponding author on reasonable request.

## Abbreviations

CRC: Colorectal cancer
CRA: Colorectal adenoma
